# MRP8/ABCC11 Expression Is Regulated by Dexamethasone in Breast Cancer Cells and Is Associated to Progesterone Receptor Status in Breast Tumors

**DOI:** 10.4061/2011/807380

**Published:** 2011-01-20

**Authors:** Mylène Honorat, Aurélia Mesnier, Julie Vendrell, Attilio Di Pietro, Valérie Lin, Charles Dumontet, Pascale Cohen, Léa Payen

**Affiliations:** ^1^Université de Lyon, Lyon1, Lyon 69008, France; ^2^Inserm, U590, Lyon 69008, France; ^3^Centre Léon Bérard, FNCLCC, Lyon 69008, France; ^4^Equipe Labellisée Ligue 2009, Institut de Biologie et Chimie des Protéines FR 3302, BM2SI, UMR 5086/Université Lyon 1, IFR128 BioSciences Gerland, 69367 Lyon Cedex 07, France; ^5^School of Biological Sciences, Nanyang Technological University, Singapore 637616; ^6^Laboratoire de Toxicologie, Faculté de Pharmacie, ISPB, Université de Lyon, Lyon 69008, France; ^7^Hospices Civils de Lyon, Centre Hospitalier Lyon-Sud, Laboratoire de biochimie, 69495 Pierre-Bénite, France

## Abstract

The ATP-binding cassette multidrug resistance protein 8 (MRP8/ABCC11) mediates the excretion of anticancer drugs. ABCC11 mRNA and protein levels were enhanced by DEX (dexamethasone) and by PROG (progesterone) in MCF7 (progesterone receptor-(PR-) positive) but not in MDA-MB-231 (PR-negative) breast cancer cells. This suggested a PR-signaling pathway involvement in ABCC11 regulation. Nevertheless, pregnenolone-16**α**-carbonitrile (GR antagonist) and clotrimazole strongly and moderately decreased ABCC11 expression levels in Glucocortocoid Receptor-(GR-) and Pregnane X Receptor (PXR)-positive MCF7 cells but not in MDA-MB-231 cells (GR- and PXR-positive). Thus, GR-signaling pathway involvement could not be excluded in ABCC11 regulation in MCF7 cells. Furthermore, ABCC11 levels were positively correlated with the PR status of postmenopausal patient breast tumors from two independent cohorts. Thus, in the subclass of breast tumors (Estrogen Receptor-(ER-) negative/PR-positive), the elevated expression level of ABCC11 may alter the sensitivity to ABCC11 anticancer substrates, especially under treatment combinations with DEX.

## 1. Introduction

Among the members of the ATP-binding cassette (ABC) transporter superfamily, MRP8/ABCC11 (MultiDrug Resistance Protein 8) is a full-length ABC transporter associated with resistance to methotrexate and fluoropyrimidines, two classes of agents widely used for breast cancer treatments [1, 2]. ABCC11 transcripts were overexpressed in estrogen receptor-(ER-) positive breast cancers [3]. As apically expressed, ABCC11 protein is likely to play a significant role in absorption, distribution and elimination of its substrates [4]. ABCC11-mediated transport of anticancer drugs, combined with its expression levels in a hormonally-regulated breast tissue, suggest that the pump expression may be regulated by xenobiotics. Dexamethasone (DEX), a potent anti-inflammatory factor, has already found very broad clinical applications for treatment of diverse conditions, ranging from associated diseases and asthma to cancer therapy [5]. DEX is a well-known activator of numerous signal transduction and molecular gene-regulation pathways. It frequently involves nuclear receptors, including pregnane X receptor (PXR), glucocorticoid receptor (GR) and progesterone receptor (PR) [6]. The aim of this work was to determine whether a DEX treatment may lead to any change in the expression level of ABCC11 protein in MCF7 breast cancer cells, an endocrine-related cell model.

## 2. Materials and Methods

### 2.1. Chemicals and Cell Lines

DEX, PROG, clotrimazole and pregnenolone 16*α*-carbonitrile (PCN) were purchased from Sigma Aldrich (France). TRIZol RNA extraction kit, murine moloney leukemia virus reverse transcriptase (MMLV-RT), Taq DNA polymerase, complete high-glucose Dulbecco's modified Eagle's medium (DMEM), L-glutamine and penicillin-streptomycin were manufactured by Gibco (France), and fetal bovine sera by PAN Biotech GmbH (Aidenbach, Germany). To avoid interference by steroids present in classical media, cell lines were first purged for 4 days in DMEM without phenol red, supplemented with 3% steroid-depleted, dextran-coated and charcoal-treated, fetal calf serum (DCC medium). The culture media were then used in either presence or absence of various molecules as described in Honorat et al. (2008) [3]. We used (PR-positive, ER-positive, GR-positive and PXR-positive) MCF7 and (PR-negative, ER-negative, GR-positive and PXR-positive) MDA-MB231 cells stably transfected with both empty pBK-CMV and pSG5-plasmids (called pSG5-MDA-MB231). The last cell line is derived from MDA-MB-231 cells and displays a similar phenotype about PR and ER status.

### 2.2. Breast Tumor Samples

All experimental procedures were performed in compliance with French laws and regulations and were approved by the Ethics Committee. Sixty primary tumors from patients, with no therapy prior to surgery, were collected from postmenopausal women at the Pathology Department of Val d'Aurelle Cancer Center (Montpellier, France) [7]. The malignancy of infiltrating carcinomas was scored according to the histological prognostic system of Scarff-Bloom-Richardson [8]. The ER-positive status was determined at the protein level by a radio-ligand binding assay (with a cut-off level for positivity set at 10 fmol/mg of protein according to the WHO typing system) and confirmed by an ER quantitative RT-PCR assay. The PR status was measured at the protein level by a binding assay as previously described [9]. 

In parallel, normalized data from an independent cohort of 245 breast tumors (Bittner_Breast's study) were extracted from the GEO website. ABCC11 expression levels were analyzed in breast tumors whose PR and ER clinical status were indicated (mostly defined by immunohistochemistry (IHC)).

### 2.3. Quantitative Real-Time RT-PCR (QRT-PCR)

Total mRNA extraction and QRT-PCR were performed as described in Honorat et al. (2008) [3].

### 2.4. Immunoblotting and Quantification

Protein expression was analysed by immunoblotting analysis as previously described in [3]. Densitometry was performed using the ImageJ software.

### 2.5. Statistical Analysis

Data were analyzed for statistical significance using either Mann Whitney's test or Student's *t*-test. Differences with *P*-values <.05 were considered as statistically significant.

## 3. Results and Discussion

**Figure 1:**
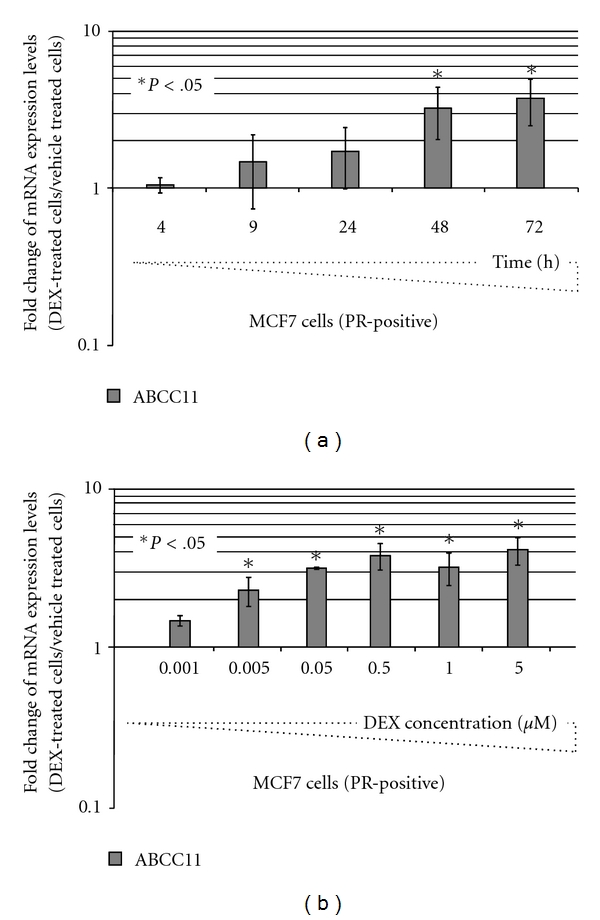
DEX time-course and dose-dependent effects on ABCC11 mRNA expression. MCF7 cells were exposed to 5 *μ*M DEX for 4 to 72 h (a) or to DEX concentrations ranging from 0.001 to 5 *μ*M (b). Fold change of mRNA levels of ABCC11 was determined by QRT-PCR. The QRT-PCR values indicated below are means ± S.D. of at least 4 independent experiments; *y* is a logarithmic scale. *P < .05; student's *t*-test.

**Figure 2:**
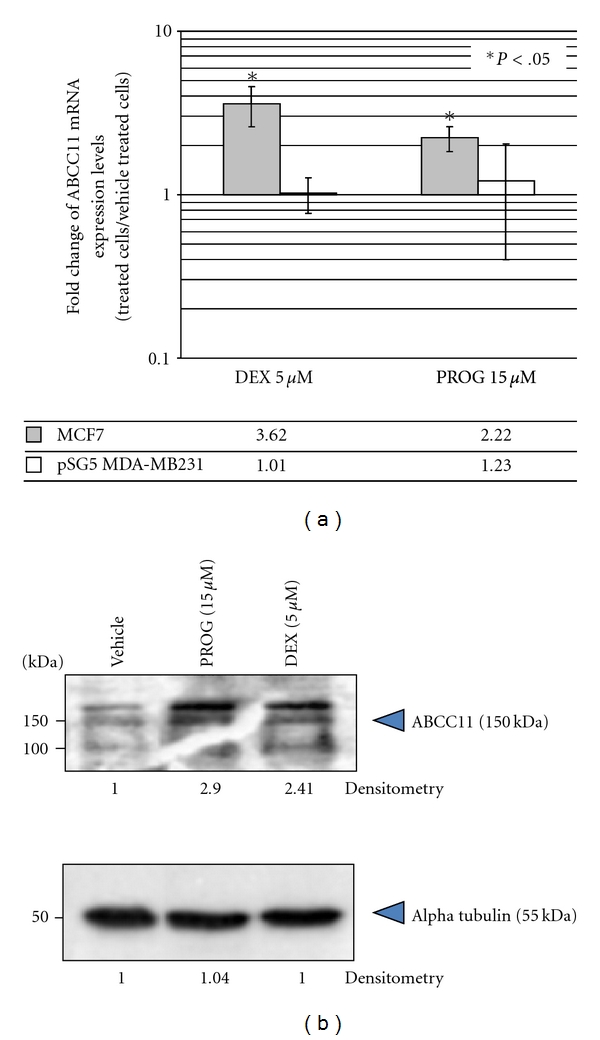
Alterations of ABCC11 mRNA and protein level by DEX and PROG. (a) Cells were exposed to either 5 *μ*M DEX or 15 *μ*M PROG for 72 h. Fold change of mRNA levels of ABCC11 was determined by QRT-PCR. The QRT-PCR values indicated below are means ± S.D. of 4 independent experiments; *y* is a logarithmic scale. *P < .05; student's *t*-test. (b) Crude membrane fractions (150 *μ*g) were prepared from cells exposed to either vehicle, 5 *μ*M DEX or 15 *μ*M PROG for 72 h.

The expression levels of ABC transporters are highly regulated by xenobiotics, including estrogens, PROG and DEX [2]. These regulations may control the availability of many substrates, including anticancer drugs, by either increasing or decreasing their elimination from cells. They can influence the cell sensitivity to their substrates. Consequently, we characterized DEX regulation pathways of *ABCC11* in PR-positive MCF7 cells. For the first time, ABCC11 expression levels were shown to be increased in a time-dependent manner by 5 *μ*M DEX, with a trend towards higher ABCC11 levels after a 24-h exposure, and a maximal and significant induction after 48–72 h (Figure 1(a)) in DEX-treated cells compared to vehicle-treated cells. Furthermore, DEX strongly enhanced ABCC11 mRNA levels in a dose-dependent manner (Figure 1(b)). ABCC11 mRNA amounts were slightly upregulated after 48-h exposure to a very low DEX concentration (0.001 *μ*M, Figure 1(b)); higher concentrations (0.05 *μ*M) were however required to reach a maximal and significant induction. The molecular mechanisms of *ABCC11* regulation were further explored by demonstrating that, in MCF7 cells, the physiological PR ligand PROG regulated ABCC11 expression in the same way as DEX, while DEX and PROG did not modify ABCC11 expression in PR-negative pSG5-MDA-MB-231 breast cells. PROG at 15 *μ*M induced *ABCC11* gene expression in MCF7 cells, whereas no regulation was observed in PR-negative pSG5-MDA-MB-231 cells (Figure 2(a)). In agreement, by immunoblotting, both DEX and PROG increased ABCC11 protein expression, by at least 2-fold, in comparison to the vehicle control (Figure 2(b)). Additional bands, observed below 116 kDa, were probably due to proteolytic degradation of ABCC11 as described by Bortfeld et al. [4]. DEX can activate PR-signaling pathways [6, 10]. Since ABCC11 regulation by DEX and PROG only occurred in PR-positive MCF7 cells and not in PR-negative pSG5-MDA-MB-231 cells, we hypothesized that the effects of DEX and PROG on ABCC11 expression could be partially related to PR-signaling pathways. Interestingly, by *in silico* analysis of the human *ABCC11* promoter region (−5000, chr16: 38685979-), we found two progesterone-response elements (PRE).

**Figure 3:**
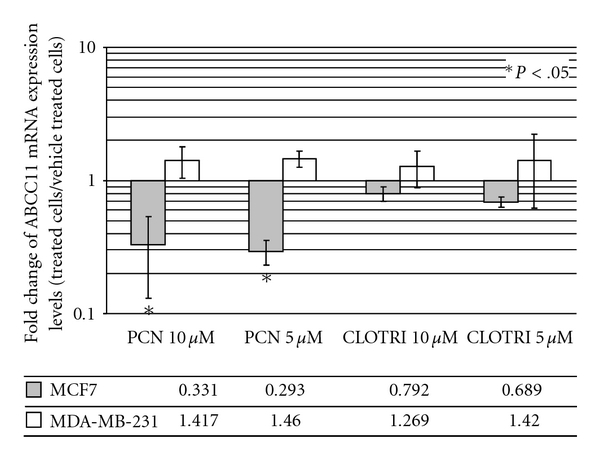
Alteration of ABCC11 mRNA level by clotrimazole and PCN. MCF7 cells were treated for 72 h with 15 *μ*M PROG, 5–10 *μ*M clotrimazole or 5–10 *μ*M PCN. Fold change of mRNA levels of ABCC11 was determined by QRT-PCR. The QRT-PCR values indicated below are means ± SD. at least three independent experiments. *P < .05; student's *t*-test.

Based upon the observation that ABCC11 expression levels were upregulated by DEX, we next evaluated whether ABCC11 expression was also regulated by other ligands of GR and PXR. PCN is known as a weak hPXR activator and as a potential GR antagonist [11–13]. PCN at 5 *μ*M and 10 *μ*M strongly decreased ABCC11 expression in MCF7 cells and had no effect in MDA-MB-231 cells (Figure 3). Though MDA-MB-231 cells strongly expressed GR, no effects of either DEX or PROG were observed on ABCC11 expression (Figure 2(a)). The default of PCN, DEX and PROG effects via GR in MDA-MB-231 cells may be directly related to the “cellular context”. Indeed, GR may regulate gene transcription by various mechanisms: synergistic activity where transcription factors bound at other DNA sites cooperate with ligand-activated GR bound to a consensus glucocorticoid-response element (GRE); interference with the actions of other transcription factors (e.g., AP-1 and NF-*κ*B) through protein-protein interactions, which does not require GR binding to DNA; and either repression or stimulation via requisite interactions of GR with other transcription factors all bound at so-called composite GREs [14–16]. DEX had been demonstrated to increase c-fms transcript and protein levels in breast cells (SKBR3 and BT20), but not in others (MCF7) [17]. Consequently, in MCF7 cells, the involvement of GR-signaling pathways could not be completely excluded and is somehow suggested by the GRE motif revealed in the *in silico* analysis of the human *ABCC11 *promoter region (−5000) using the Genomatix software.

Finally, we used clotrimazole, a powerful human PXR ligand to explore PXR signaling pathways. Clotrimazole at 5–10 *μ*M did not produce a major effect on *ABCC11* expression in MCF7 cells and had no effect on ABCC11 expression in MDA-MB231 cells (Figure 3). In addition, little is known about PROG direct interaction with PXR-signaling pathway, and PCN and DEX were relatively weak activators of human PXR as compared to clotrimazole. Although PCN weakly decreased *ABCC11* expression in PXR-positive MCF7 cells, no regulatory effect was observed in PXR-positive MDA-MB-231 cells. We may then suggest that PXR-signaling pathway was likely not directly involved in ABCC11 regulation by DEX and PROG. The overall results suggest that regulation of ABCC11 expression by PROG and DEX is at least partially linked to PR signaling pathways.

Currently, the PR-positive status of breast tumors is recognized as an important indicator of the likelihood of response to endocrine agents [18]. Approximately two-thirds of breast cancers express the estrogen receptor alpha (ER*α*), some of them being PR-negative. PR absence significantly correlated with a less differentiated phenotype of breast tumors [18]. Based upon our *in vitro* observation, we evaluated whether there was any correlation between ABCC11 expression and PR status in 60 breast cancers from postmenopausal women (fully described [3, 7]). In this local cohort of patient, the PR and ER status were evaluated at the protein level by binding assays [7]. Significantly higher ABCC11 mRNA levels were observed in PR-positive tumors than in PR-negative samples (ABCC11 levels were approximately 6-fold higher in the PR-positive groups than in the PR-negative groups; *P* = .014 Mann Withney's test (Table 1)). To confirm those observations, we extracted and analysed the normalized data of ABCC11 expression level of 245 breast tumors from an independent cohort (Bittner_Breast's study from the GEO website). We only retained breast tumors whose PR and ER clinical status, mostly defined by immunohistochemistry (IHC), were indicated. We validated a positive association between PR status and ABCC11 expression levels in this larger cohort of breast tumors (Table 1; *P* = .003). 

We previously observed a high significant *ABCC11* mRNA levels in breast cancer tumors possessing high levels of ER*α* compared to those having low levels [3]. We validated a positive association between ABCC11 expression level and ER status in Bittner's breast cohort (*n* = 159; ER-negative tumors; *n* = 86; ER-positive tumors; *P* = .0004 Mann Whitney's test). We decided to carry out subclasses of tumors according their ER status. In our tumor cohort, the ABCC11 mRNA level difference between breast tumors according to PR-status was only found significant in patients with ER-negative status. ABCC11 expression levels were approximately 11-fold higher in the ER−/PR+ subgroup than in ER−/PR− patient subgroup (*P* = .009; Mann Whitney's test). While not reaching significant difference (*P* = .076), a tendency had been found in the ER-negative subgroup of Bittner's Breast cohort. The median ratio between PR−/PR+ was approximately 9-fold (Table 1). This might be directly related to the tiny number of ER−/PR+ tumors (*n* = 3). Since this repartition is representative of clinical situation (ER-positive status is frequently associated with PR-positive status), this observation fully justify the limited number of ER−/PR+ tumors in studied clinical cohorts.

By contrast, in both cohorts, tumors with ER-positive status did not show any correlation between ABCC11 expression levels and PR status (Table 1). This suggests that in ER-positive breast tumors, the ER status is mainly associated in *ABCC11* mRNA levels, while in ER-negative breast tumors ABCC11 expression levels are associated with PR-status.

## 4. Conclusion

Currently, breast chemotherapies frequently include 5-FU (metabolized in cells to 5FdUMP, which is an ABCC11 substrate). Interestingly, Park et al. studied the relationship between ABC transporter gene expression and chemotherapy responsiveness in early breast cancer patients who underwent sequential weekly paclitaxel/FEC (5-FU, epirubicin and cyclophosphamide) neoadjuvant therapy. Several ABC transporters including ABCC5 and ABCC11 showed significant increased expression in the residual disease [19]. Taken altogether, the association between PR and ABCC11, in a subgroup of breast tumors with low expression of ER, highlighted how ABCC11 might constitute a putative marker of 5-FU anticancer resistance.

Furthermore, by altering ABCC11 expression levels, DEX, an adjuvant anticancer drug, may significantly contribute to chemo-resistance mechanisms to ABCC11 substrates. High expression levels of ABCC11 in PR-positive breast tumors with low expression of ER alpha may contribute to a decreased sensitivity to chemotherapeutic combinations containing 5-FU. Future work is needed to investigate this point.

## Figures and Tables

**Table 1:** Relationships between ABCC11 mRNA expression, PR-status and ER-status in breast tumors from postmenopausal patients.

	Subgroup	*n*	Median	Range (min-max)	Ratio (subgroup 2 median/subgroup 1 median)	*P* ^*‡*^
Present study^†§^	PR−	26	**2,22**	(0,1–289)	5,79	**.014**
	PR+	34	**12,86**	(0,6–354)		
	ER−/PR−	23	**1,50**	(0,1–177)	11,21	**.009**
	ER−/PR+	12	**16,82**	(0,8–354)		
	ER+/PR−	3	**33,37**	(8,9–289)	0,36	.181
	ER+/PR+	22	**11,99**	(0,6–286)		

Bittner's study	PR−	116	**86,50**	(2,4–12494)	2,47	**.003**
	PR+	129	**213,50**	(1,7–11933)		
	ER−/PR−	83	**53,80**	(2,4–12494)	9,80	.075
	ER−/PR+	3	**527,10**	(199,8–1866)		
	ER+/PR−	33	**225,10**	(4,6–4950)	0,87	.871
	ER+/PR+	125	**196,00**	(1,7–11933)		

Present study: *n* = 60 tumors.

Bittner's study: *n* = 245; from normalized data published on GEO website GSE2109-ABCC11 (224146_s_at).

^†^ABCC11 QRT-PCR expression levels.

^§^PR and ER status were measured at the protein level by binding assay.

^‡^ *P*-values were considered to be statistically significant if *P* < .05 (Mann Whitney's test).
